# Selenomethionine protects hematopoietic stem/progenitor cells against cobalt nanoparticles by stimulating antioxidant actions and DNA repair functions

**DOI:** 10.18632/aging.202865

**Published:** 2021-04-19

**Authors:** Wenfeng Zhu, Yake Liu, Weinan Zhang, Wentao Fan, Siqi Wang, Jin-Hua Gu, Huanjian Sun, Fan Liu

**Affiliations:** 1 Orthopaedic Laboratory, Affiliated Hospital of Nantong University, Nantong, Jiangsu Province, China; 2 Department of Orthopaedics, Affiliated Hospital of Nantong University, Nantong, Jiangsu Province, China; 3 Department of Clinical Pharmacy, Affiliated Maternity and Child Health Care Hospital of Nantong University, Nantong, Jiangsu Province, China; 4 Department of Orthopaedics, The Sixth Affiliated Hospital of Nantong University, Yancheng, Jiangsu Province, China

**Keywords:** selenium, hematopoietic stem/progenitor cells, cobalt nanoparticles, antioxidant action, DNA repair function

## Abstract

Hematopoietic stem cells (HSCs) and hematopoietic progenitor cells (HPCs) can differentiate into all blood lineages to maintain hematopoiesis, wound healing, and immune functions. Recently, cobalt-chromium alloy casting implants have been used extensively in total hip replacements; however, cobalt nanoparticles (CoNPs) released from the alloy were toxic to HSCs and HPCs. We aimed to investigate the mechanism underlying the toxic effect of CoNPs on HSCs/HPCs and to determine the protective effect of selenomethionine (SeMet) against CoNPs *in vitro* and *in vivo*. Human and rat CD34+ HSCs/HPCs were isolated from cord blood and bone marrow, respectively. CoNPs decreased the viability of CD34+ HSCs/HPCs and increased apoptosis. SeMet attenuated the toxicity of CoNPs by enhancing the antioxidant ability of cells. The protective effect of SeMet was not completely abolished after adding H2O2 to abrogate the improvement of the antioxidant capacity by SeMet. SeMet and CoNPs stimulated ATM/ATR DNA damage response signals and inhibited cell proliferation. Unlike CoNPs, SeMet did not damage the DNA, and cell proliferation recovered after removing SeMet. SeMet inhibited the CoNP-induced upregulation of hypoxia inducible factor (HIF)-1α, thereby disrupting the inhibitory effect of HIF-1α on breast cancer type 1 susceptibility protein (BRCA1). Moreover, SeMet promoted BRCA1-mediated ubiquitination of cyclin B by upregulating UBE2K. Thus, SeMet enhanced cell cycle arrest and DNA repair post-CoNP exposure. Overall, SeMet protected CD34+ HSCs/HPCs against CoNPs by stimulating antioxidant activity and DNA repair.

## INTRODUCTION

An increasing number of patients with end-stage osteoarthritis of the hip or osteonecrosis of the femoral head are undergoing total hip replacement (THR) surgery to manage their diseases. Cobalt-chromium (CoCr) alloy casting implants have been used extensively in THR over the last decade because they have better wear characteristics than polyethylene—previously used in conventional THR—but was limited by osteolysis and late aseptic loosening. However, in certain clinical settings, THR using CoCr alloy casting implants has been associated with several adverse reactions [1, 2]. These adverse reactions may be triggered by wear products (i.e., metal particles, <50 nm in size) that are generated at the articulation. Cobalt nanoparticles (CoNPs) are the most common wear products. CoNPs are incorporated into the periprosthetic tissue or enter the bloodstream, and reach organs and tissues, such as the heart, liver, brain, kidney, and bone marrow of patients [3–5]. Furthermore, several *in vitro* studies have shown that CoNPs can enter cells and induce oxidative stress, DNA damage, and genotoxicity [6, 7].

Hematopoietic stem cells (HSCs) are multipotent stem cells that give rise to erythrocytes, megakaryocytes/platelets, and various types of immune cells, such as monocytes, macrophages, neutrophils, eosinophils, basophils, T and B lymphocytes, and natural killer cells. Therefore, any imbalance in the levels of HSCs can affect hematopoiesis, wound healing, and immune functions, and even result in the development of hematopoietic malignancies such as leukemia [8–11]. Although HSCs reside primarily in the bone marrow, they are also found in peripheral blood and are abundant in cord blood. HSCs can self-renew to maintain an adequate pool of hematopoietic cells, or differentiate into hematopoietic progenitor cells (HPCs). HPCs undergo massive proliferative expansion to meet the needs of hematopoiesis on a daily basis or in response to stress. A previous study reported that CoNPs exert a toxic effect on HSCs and HPCs [12], but the underlying mechanism was unclear.

The conservation and integrity of DNA is crucial for the viability and fitness of all living cells and organisms. However, DNA is easily damaged by both endogenous and exogenous agents, such as reactive oxygen species (ROS), heavy metals, and radiation. To maintain genomic stability, cells have evolved DNA repair mechanisms that involve important cellular processes, such as activation of DNA repair pathways, cell cycle arrest, DNA repair, and cell survival or apoptosis. Post-translational modification of cellular proteins by ubiquitination plays numerous roles in coordinating and co-regulating DNA repair functions [13, 14]. Numerous DNA repair factors are subject to ubiquitylation or deubiquitylation during the DNA repair processes. For example, RNF8 promotes histone ubiquitylation, which leads to the recruitment of several DNA repair factors, including breast cancer type 1 susceptibility protein (BRCA1) and 53BP1, to the break sites [15].

Selenium (Se) is an essential trace element that regulates various cellular processes. Se is present at the active site of diverse antioxidant enzymes such as glutathione peroxidases (GPxs) and thioredoxin reductases; therefore, supplementation with Se improves the antioxidant status when ROS are overproduced in oxidative stress-related diseases [16, 17]. In addition, increasing evidence suggests that Se maintains genome integrity by activating the DNA repair pathways [18, 19]. For instance, pretreatment with low doses of Se protects against ultraviolet-induced genotoxicity [19]. Proteomic analysis revealed that both inorganic and organic Se increased the expression of ubiquitin-conjugating enzyme E2 (UBE2K) in colon cells [20]. An interaction between UBE2K and BRCA1 is a prerequisite for the synthesis of Lys48-linked polyubiquitin chains [21]. Through this mechanism, BRCA1 induces the ubiquitination and degradation of RNA polymerase II and cyclin B, and plays a role in homologous recombination during DNA repair [22, 23]. Based on these findings, we hypothesize that Se promotes DNA repair by activating the UBE2K-BRCA1 pathway in HSCs/HPCs upon exposure to CoNPs. Thus, this study aims to elucidate the mechanism by which Se attenuates CoNP toxicity in HSCs/HPCs.

## MATERIALS AND METHODS

### Isolation of human CD34^+^ HSCs/HPCs from cord blood

Human cord blood samples were collected from umbilical cord blood vessels present in the placentas of full-term infants at the Affiliated Hospital of Nantong University (Nantong, China). Informed consent was obtained from the mothers, and the study was approved by the Ethics Committee of the Affiliated Hospital of Nantong University. Mononuclear cells were separated from cord blood via density gradient centrifugation (Ficoll-Paque™ Plus, GE Healthcare, Uppsala, Sweden). Subsequently, CD34^+^ HPCs were isolated using the EasySep® Human CD34 Positive Selection Kit (Stem cell Technologies, Grenoble, France) according to the manufacturer’s guidelines.

### Isolation of rat CD34^+^ HSCs/HPCs from bone marrow

The femurs and tibia of rats were dissected with scissors at the joints of both ends. The marrow cavity was washed repeatedly with the Dulbecco’s modified Eagle’s medium (DMEM; GIBCO, Shanghai, China). An 18-gauge needle was gently passed through the marrow cavity to obtain dissociated cells. The cells were used to isolate mononuclear cells and CD34^+^ HSCs/HPCs using the methods described above. All the animal studies were approved by the ethical commission of the Affiliated Hospital of Nantong University.

### *Ex vivo* expansion of CD34^+^ HSCs/HPCs

Purified CD34^+^ HPCs were cultured in DMEM supplemented with 10% fetal bovine serum (GIBCO). Recombinant cytokines, including stem cell factor, Flt-3 ligand, and thyroperoxidase were purchased from Stem Cell Technologies (Vancouver, BC, Canada) and were used at concentrations of 100 ng/mL. The cells were incubated in a fully humidified incubator at 37° C in an atmosphere containing 5% CO_2_.

### Preparation of cell medium containing CoNPs

CoNPs (50–200 nm) were purchased from Fluka Chemical (Seltzer, Germany). CoNPs were weighed and depyrogenated at 189° C for 90 min in glass vials. Dry sterilized NPs were resuspended by drop-wise addition in the culture medium and immediately mixed by vortexing. The NPs were then sonicated in a bath sonicator for 15 min using an ultrasonic oscillator (Ningbo Sklon Lab Instrument; Shanghai, China). Vortexing and sonication were repeated before the treatment of cells with NPs in each experiment.

### Cell treatments

Based on the results from our pilot experiments, CoNP concentrations ranging from 0‒1000 μM were used. Human and rat CD34+ HSCs/HPCs were cultured in medium containing CoNPs. Various concentrations of Na2SeO3, selenomethionine (SeMet), and selenocysteine (SeCys) were added to the cells 15 h before CoNP treatment to determine their protective effects. Cells underwent all measurements 24 h after the treatments.

H_2_O_2_ was purchased from Sigma-Aldrich (Shanghai, China), and 1 μM was added to the cells to neutralize the anti-oxidative capacity induced by SeMet. An inhibitor of mutated ataxia telangiectasia (ATM) and Rad3-related (ATR), CGK773, and an activator of hypoxia inducible factor (HIF)-1α, dimethyloxalyglycine, were purchased from Selleck Chemicals (Shanghai, China). Cells were treated with CGK773 (200 nM) and dimethyloxalyglycine (5 μM) to suppress ATM/ATR and stimulate HIF-1α signals, respectively.

BRCA1, UBE2K, and HIF-1α were knocked down by transfecting cells with small interfering (si)RNA-BRCA1, siRNA-UBE2K, and siRNA-HIF-1α, respectively. These siRNAs were synthesized by GenePharm (Shanghai, China). All siRNAs and the scrambled siRNA (a non-targeting siRNA) were transfected into cells using a Lipofectamine 2000 kit (Invitrogen, Carlsbad, CA, USA) following the manufacturer's protocol. For overexpression of UBE2K, the coding region was cloned into the enhanced green fluorescent protein plasmid-C1 vector (GenePharma) to construct an overexpression vector. Lipofectamine 2000 was also used in the transfection of this vector.

### 3-(4,5-Dimethylthiazol-2-yl)-2,5-diphenyltetrazolium bromide (MTT) assay

The viability of CD34^+^ HSCs/HPCs was assessed using the MTT assay. CD34^+^ HPCs were plated into 96-well culture plates (5 × 10^3^ cells per well). After the above-mentioned treatments, 100 μL of MTT solution (1 mg/mL) were added to each well and the cells were further incubated for 4 h at 37° C. The MTT solution was then removed and 150 μL of dimethyl sulfoxide was added to the cells to solubilize the formazan crystals. Optical density was measured at 570 nm using a microplate reader (BioRad, Hercules, CA, USA) and correlated with cell viability.

### Flow cytometric assessment of apoptosis and cell cycle analysis

An apoptosis detection kit provided by Beyotime Biotechnology (Shanghai, China) was used to detect and quantify apoptosis in vascular endothelial cells. Briefly, cells were trypsinized and resuspended at a concentration of 1 × 10^6^/mL in diluted binding buffer, then labeled with 10 μL of Annexin V-fluorescein isothiocyanate. Cells were incubated for 30 min at room temperature in the dark, followed by a 5 min incubation with 5 μL of propidium iodide (PI). Subsequently, 400 μL of 1× binding buffer was added to each tube. Flow cytometric analysis was performed to monitor Annexin V and DNA-bound PI. Data acquisition and analysis were performed using FlowJo software.

For cell cycle analysis, cells were fixed with 70% chilled ethanol at 4° C overnight. The cells were rehydrated, washed twice with ice-cold phosphate-buffered saline (PBS), and incubated with 10 μg/mL RNase (Fermentas, Shanghai, China) at 37° C. Subsequently, the cell cycle was observed via PI staining of the nuclei and analyzed using FACS flow cytometry (BD Biosciences, Franklin Lakes, NJ, USA).

### Detection of ROS and 8-hydroxydeoxyguanosine (8-OHdG)

Intracellular ROS levels were evaluated using 2,7-dichlorofluorescin diacetate (DCFH-DA) (Beyotime Biotechnology). DCFH-DA can form the fluorescent compound, dichlorofluorescein, in the presence of ROS. First, cells were incubated with DCFH-DA (10 μM) for 20 min at 37° C; then, cells were washed at least five times with serum-free medium. Labeled cells were trypsinized, resuspended in PBS supplemented with 5% fetal bovine serum, and analyzed by flow cytometry (BD Biosciences). A minimum of 10,000 cells were analyzed per condition.

The level of 8-OHdG in cells was detected using an ELISA kit (Shanghai Yuanye Bio-Technology, Shanghai, China) as per the manufacturer's instructions. Concentrations were determined by comparing the optical density values to a standard curve.

### Detection of total antioxidant capacity (T-AOC), the glutathione (GSH) level, and glutathione peroxidase (GPx) activity

After treatment, HSCs/HPCs were collected and the supernatant was obtained after sonication and centrifugation. Soluble protein concentrations were measured using the bicinchoninic acid assay. T-AOC was detected through chemical colorimetric analysis of the ferric reducing ability of cells at 593 nm using an enzymatic assay kit (Beyotime Biotechnology) according to the manufacturer’s instructions. In addition, the GSH level and GPx activity in the cells were determined using colorimetric assay kits (Beyotime Biotechnology) according to the manufacturer’s protocols.

### Comet assay

Cells (1000 cells/10 μL) were mixed with 70 μL low melting point agarose (0.5%) at 37° C, placed on fully frosted slides (Thermo Fisher Scientific, Waltham, MA, USA) coated with a thin layer of normal melting point agarose (1%) and covered with a coverslip. The slides were immersed in a cold lysis solution (2.5 M NaCl, 100 mM EDTA, 10 mM Tris, 1% Triton X-100, and 10% dimethyl sulfoxide, pH 10) at 4° C for 1 h. The slides were then placed in a horizontal electrophoresis chamber (Owl A5, Thermo Fisher Scientific) containing the cold electrophoresis alkaline buffer (300 mM NaOH, 1 mM EDTA, pH 13) and run at 25 V and 300 mA for 20 min in the dark. Next, ethidium bromide (20 mg/mL; 50 μL) was added to each slide to stain the DNA. The slides were examined under a fluorescence microscope (Leica Microsystems, Buffalo Grove, IL, USA) equipped with filters and a digital camera. Comet lengths were analyzed using CaspLab software.

### Immunofluorescence (IF) assay

Cells were fixed with 4% paraformaldehyde for 15 min and blocked with PBS containing 0.3% Triton X-100 and 5% bovine serum albumin (w/v) for 1 h at 37° C before incubation with an antibody against γH2AX (1:500; abcam, Cambridge, MA, USA). Cells were incubated with the secondary fluorescent-labeled antibody (Alexa Fluor 488, Invitrogen) in the dark prior to microscopy-based analysis.

### 5-Ethynyl-2ʹ-deoxyuridine (EdU) assay

The EdU assay was performed using a Cell-Light EdU DNA Cell Proliferation Kit (RiboBio, Shanghai, China). Human and rat CD34^+^ HSCs/HPCs (1 × 10^4^ cells/well) were seeded in a 96-well plate. After incubation with 50 μM EdU for 2 h, cells were fixed with 4% paraformaldehyde. 4',6-Diamidino-2-phenylindole (DAPI) was used to stain the DNA. Images were obtained using a fluorescence microscope, and the number of EdU-positive cells was counted.

### Electron microscopy

Cell pellets were fixed in 0.1μM sodium phosphate buffer and 2% glutaraldehyde, and embedded in Epon resin with an Epoxy Embedding Medium Kit (Sigma-Aldrich) following the manufacturer’s instructions. Ultrathin sections were observed under a Field Emission Gun-Environmental Scanning Electron Microscope (Quanta 200, FEI Company, Eindhoven, The Netherlands) in STEM mode.

### Western blot

Cells were lysed on ice for 30 min using a lysis buffer (Thermo Fischer Scientific). The lysate supernatants were denatured, then separated on a 4–12% bis Tris gel (Invitrogen). Proteins in the gel were then transferred to a nitrocellulose membrane and probed with anti-phosphorylated (p)-ATM (Ser-1981, 1:500 dilution), anti-p-ATR (Ser-428, 1:500 dilution), anti-ATM (1:500 dilution), anti-ATR (1:1000 dilution), anti-p-p53 (Ser-392, 1:500 dilution), anti-p53 (1:500 dilution), anti-BRCA1 (1:500 dilution), anti-HIF-1α (1:250 dilution), anti-UBE2K (1:500 dilution), anti-cyclin B (1:1000 dilution), and anti-β-actin (1:1000 dilution) primary antibodies overnight at 4° C. All antibodies were purchased from Abcam. Membrane-bound primary antibodies were detected using appropriate secondary antibodies. Equal loading of protein was ensured by measuring β-actin expression.

### Co-immunoprecipitation (Co-IP) assay

Whole cell extracts were prepared in IP lysis buffer containing 10 mM Tris-HCl, 5 mM EDTA, 50 mM NaCl, 50 mM NaF, and 1% Triton X 100 supplemented with the complete protease inhibitor cocktail and PhosStop tablets (Roche Diagnostics, Indianapolis, IN, USA). The lysates (0.5-1.5 mg) were incubated with 2 μg antibodies targeting HIF-1α, BRCA1, and cyclin B protein, as well as Protein G-Sepharose (GE Healthcare). The immunoprecipitated proteins were subjected to western blotting to determine their enrichment of ubiquitin.

### Exposure of rats to CoNPs and administration of SeMet

This study was approved by the Ethics Committee of the Affiliated Hospital of Nantong University. Male Sprague Dawley rats were 6-8 weeks old and weighed approximately 350 g at the start of the experiments. The animal study was performed according to the methods described by Brown et al. [24] and Chattopadhyay et al. [25] with minor modifications. CoNP particles were suspended in a vehicle of 1:1 rat serum: PBS (Oxoid) by sonication. A 50 μL suspension of CoNPs (1000 μg/kg body weight) was injected into the right hip joint. Rats were given three injections of CoNPs at three week intervals rather than being exposed continuously to these particles, or subjected to a higher number of procedures, for ethical reasons. Sham-treated rats received 50 μL of vehicle alone. In addition, rats received an oral dose of SeMet (2 mg SeMet/kg body weight/day) according to methods described in previous studies [26, 27]. All rats were sacrificed three weeks after the final injection of CoNPs or vehicle alone. Bone marrow was collected according to the method described above for analysis of biochemical parameters, including T-AOC, the GSH level, GPx activity, and 8-OHdG levels, and for comet and flow cytometric assays. Blood samples were collected by cardiac puncture for routine blood examination using an automatic blood cell analyzer (MC-600; iCubio, Shenzhen, China).

### Flow cytometry

Flow cytometry was used to detect the number of CD34^+^ HSCs/HPCs in rat bone marrow. Bone marrow cells were suspended in PBS supplemented with 1% fetal calf serum and an Alexa Fluor 700-labeled anti-CD34 antibody (Thermo Fisher Scientific, Shanghai, China). Flow cytometric analyses were performed on fresh cell suspensions immediately after the final collection. CD34^+^ HSCs/HPCs were identified by light scattering using gates to exclude dead cells and cell debris. For each sample, 10,000 events were acquired in the gated region. Light scattering profiles and fluorescence histograms were evaluated using CellQuest™ (BD Biosciences) and home-written MATLAB routines (The Mathworks, Eindhoven, The Netherlands). Nanoparticle loading was evaluated based on the geometric mean of the fluorescence intensity distributions. The same photomultiplier voltages and compensation settings were applied for identification of CD34^+^ HSCs/HPCs for comparison.

### Statistical analyses

Results are expressed as means ± SD of triplicate of independent experiments. Data were analyzed statistically by t-tests or a one-way analysis with Tukey’s post-hoc test using SPSS software, version 21.0 (IBM, Chicago, IL, USA). A *p* < 0.05 was considered to indicate a statistically significant difference.

## RESULTS

### Se attenuated the toxic effect of CoNPs on human and rat CD34^+^ HSCs/HPCs

**Figure 1:**
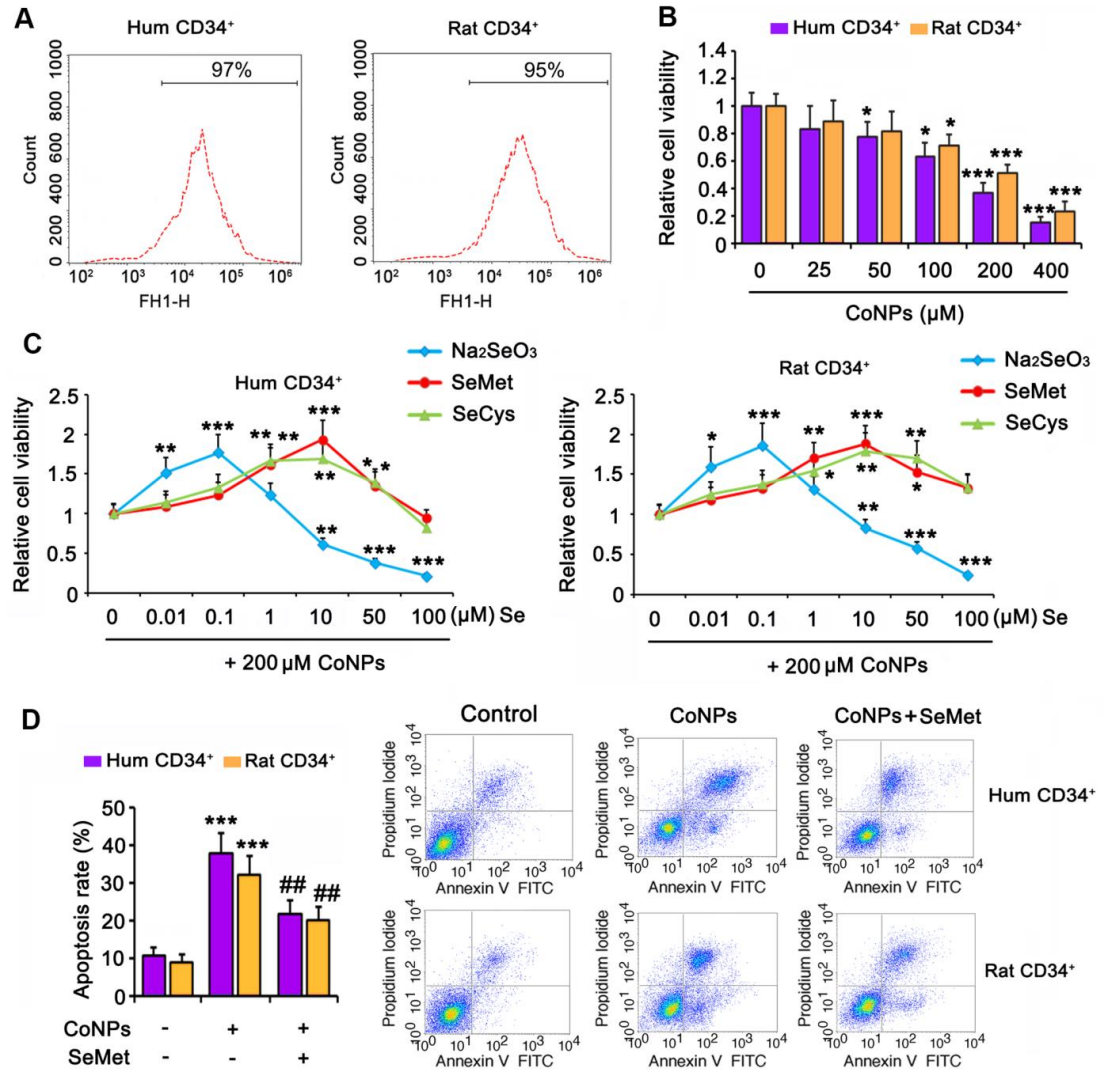
**Se attenuated toxic effect of CoNPs on human and rat CD34^+^ HSC/HPCs.** (**A**) Human and rat CD34^+^ HSC/HPCs were isolated from cord blood and bone marrow, respectively, through flow cytometry. (**B**) CD34^+^ HSC/HPCs were exposed to CoNPs at concentrations from 0 to 400 μM for 24 h, followed by MTT assay. (**C**) Different dosages of Na_2_SeO_3_, SeMet and SeCys were added to CD34^+^ HSC/HPCs 15 h before treatment with 200 μM CoNPs for additional 24 h. MTT assay was conducted to assess the cell viability. (**D**) CD34^+^ HSC/HPCs were treated with 10 μM SeMet for 15 h and then subjected to 200 μM CoNPs for 24 h. Flow cytometry was conducted to evaluate apoptosis rate. *p < 0.05, **p < 0.01, and ***p < 0.001 vs. control cells that did no subjected to any treatments; ^##^*p <* 0.01 vs. cells treated with CoNPs alone.

Human and rat CD34^+^ HSCs/HPCs were isolated from cord blood and bone marrow, respectively, by flow cytometry (Figure 1A). The isolated cells were exposed to CoNPs at concentrations ranging from 0 to 400 μM for 24 h. As indicated by the MTT assay, 50 and 100 μM CoNPs significantly decreased the viability of both human and rat CD34^+^ HSCs/HPCs (*p* < 0.05). Treatment of human and rat CD34^+^ HSCs/HPCs with 200 μM CoNPs decreased cell viability to approximately 50% compared to the control treatment *(p* < 0.001, Figure 1B). The viability of human and rat CD34^+^ HSCs/HPCs was reduced to 24.1 and 35.7%, respectively, after exposure to 400 μM CoNPs (*p* < 0.001).

To determine the effect of Se on CoNP-treated CD34^+^ HSCs/HPCs, we exposed the cells to three types of Se, namely Na_2_SeO_3_, SeMet, and SeCys, for 24 h before treatment with 200 μM CoNPs. All three Se-containing agents within the proper concentration range improved the viability of human and rat CD34^+^ HSCs/HPCs exposed to CoNPs. Cell viability was improved at the optimal concentration (0.1 μM) of Na_2_SeO_3_ (*p* < 0.001, Figure 1C). In contrast, 1 μM Na_2_SeO_3_ had no significant effect on cell viability, and Na_2_SeO_3_ at concentrations of 10, 50, and 100 μM decreased cell viability (*p* < 0.01 or *p* < 0.001). Cell viability was improved at the lowest concentration (1 μM) of SeMet and SeCys (*p* < 0.05 and *p* < 0.01, respectively). The optimal concentration of both SeMet and SeCys that improved cell viability was 10 μM. As the concentrations of both SeMet and SeCys were increased to 100 μM, improvement in cell viability was not observed. SeMet (*p* < 0.001) showed a more remarkable improvement in cell viability than SeCys (*p* < 0.01) when both were used at the optimal concentration. Although Na_2_SeO_3_ improved the viability of human and rat CD34^+^ HSCs/HPCs exposed to CoNPs, the safe concentration of Na_2_SeO_3_ was lower than that of SeMet and SeCys. Therefore, SeMet might be the most suitable Se-containing agent among these three to attenuate the toxicity of CoNPs.

In agreement with results from the MTT assay, 200 μM CoNPs caused an increase in the extent of apoptosis of human and rat CD34^+^ HSCs/HPCs (*p* < 0.001, Figure 1D). However, SeMet hindered the increase in CoNP-induced apoptosis (*p* < 0.01).

### The protective effect of SeMet against CoNP toxicity was partially associated with improved antioxidant capacity

**Figure 2:**
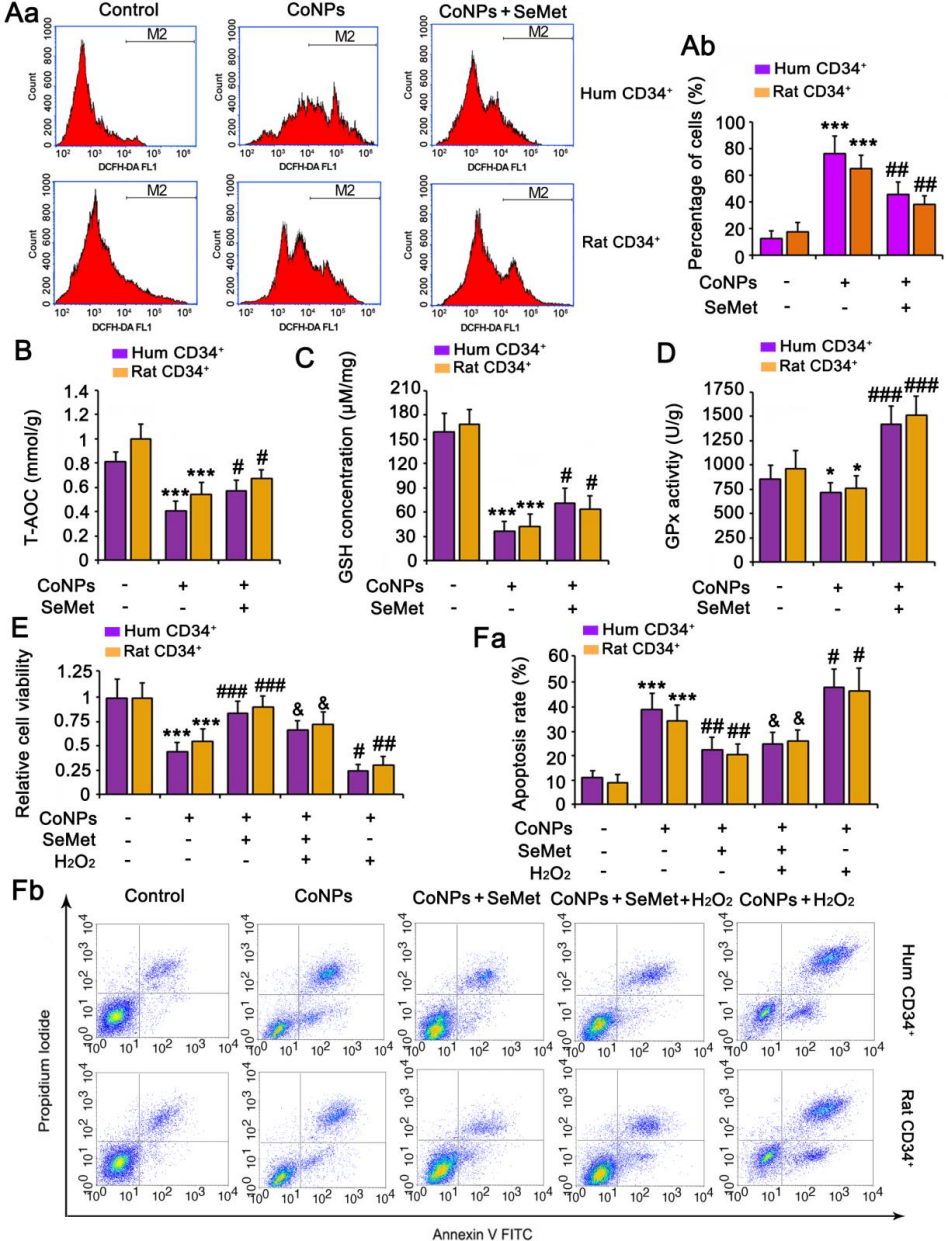
**The protective effect of SeMet against CoNPs is partially associated to the improvement of anti-oxidant capacity.** CD34^+^ HSC/HPCs were treated with 10 μM SeMet for 15 h and then subjected to 200 μM CoNPs for 24 h. Afterwards, cells underwent measurements of intracellular ROS level (**A**), T-AOC (**B**), GSH level (**C**) and GPx activity (**D**). *p < 0.05, and ***p < 0.001 vs. control cells that did no subjected to any treatments; ^#^*p <* 0.05, ^##^*p <* 0.01 and ^###^*p <* 0.001 vs. cells treated with CoNPs alone. CD34^+^ HSC/HPCs were treated with 10 μM SeMet alone or in combination with 1 μM H_2_O_2_ for 15 h. Cells were then treated with 200 μM CoNPs for 24 h, followed by measurements of cell viability (**E**) and apoptosis rate (**F**). ***p < 0.001 vs. cells treated with CoNPs alone. ^#^*p <* 0.05 vs. cells treated with SeMet and CoNPs.

Treatment with 200 μM CoNPs remarkably increased the ROS levels in human and rat CD34^+^ HSCs/HPCs (*p* < 0.001, Figure 2A). SeMet blocked the CoNP-induced increase in ROS levels (*p* < 0.01), and Se improved the antioxidant capacity of cells. Indeed, SeMet partially restored the T-AOC (*p* < 0.05, Figure 2B) and GSH level (*p* < 0.05, Figure 2C) in CD34^+^ HSCs/HPCs that were suppressed by CoNPs. Notably, SeMet increased GPx activity even in the presence of CoNPs (*p* < 0.001, Figure 2D).

To determine the role played by the antioxidative function of SeMet in protecting against CoNPs, we pretreated the cells a combination of H_2_O_2 (_to neutralize the increase in T-AOC and GSH) and SeMet, and then used CoNPs for treating the cells. As indicated by the results of cell viability and apoptosis assays, neutralizing the antioxidative effect of SeMet only partially diminished the protective effect of SeMet against CoNPs (Figure 2E, 2F).

### Both CoNPs and SeMet activated DNA damage response signals, but SeMet did not damage the DNA

**Figure 3:**
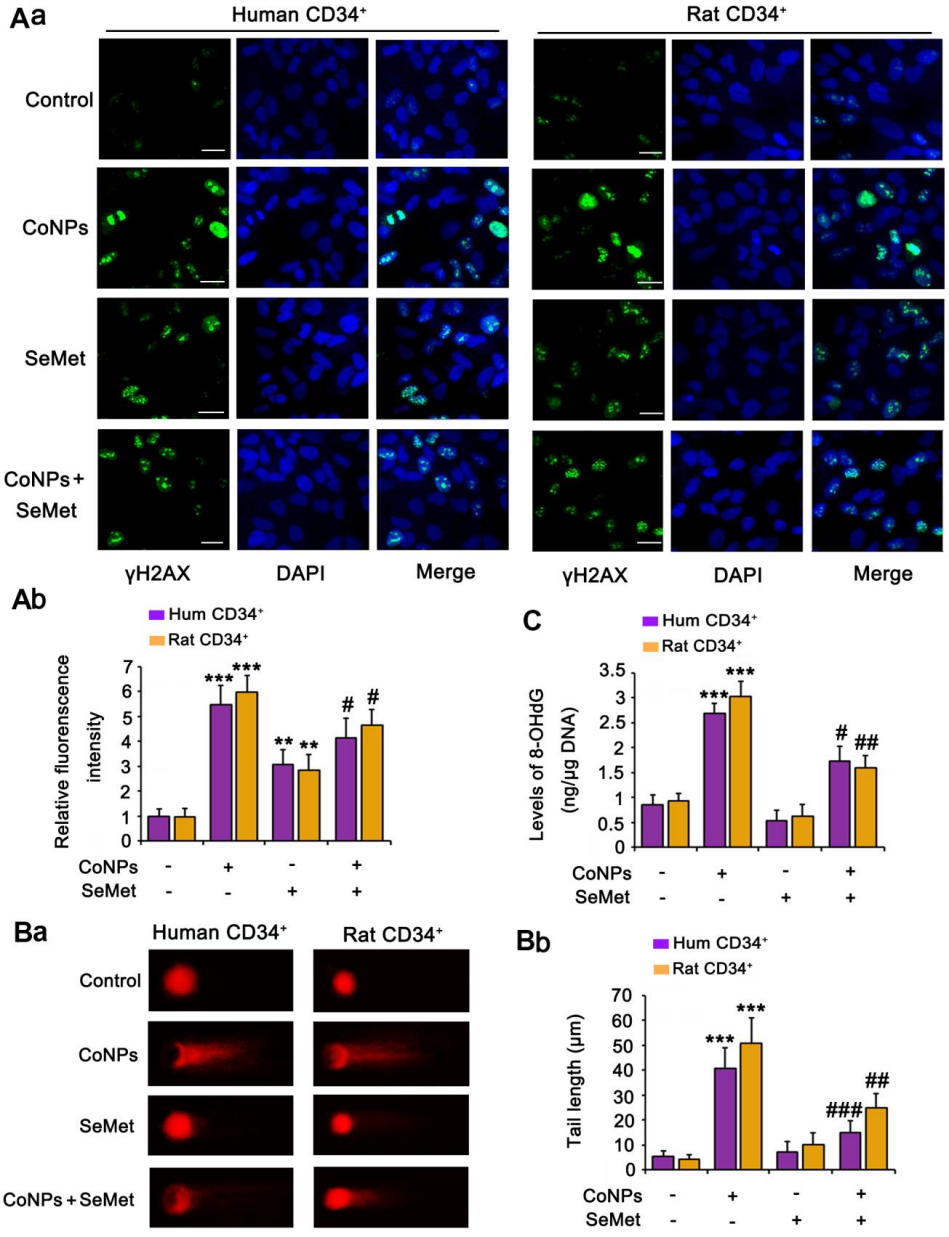
**Both CoNPs and SeMet led to the activation of γH2AX, but SeMet did not cause DNA damage.** CD34^+^ HSC/HPCs were treated with 10 μM SeMet and 200 μM CoNPs, alone or in combination. (**A**) The phosphorylation level of H2AX (γH2AX) was determined by IF analysis. DNA damage degree was evaluated by 8-OHdG level (**B**) and comet assay (**C**). ***p < 0.001 vs. control cells that did no subjected to any treatments; ^#^*p <* 0.05, ^##^*p <* 0.01 and ^###^*p <* 0.001 vs. cells treated with CoNPs alone.

Phosphorylation of H2AX (γH2AX), which is the downstream of ATM/ATR, has been considered as a sensitive marker for detecting the activation of DNA damage response signals. IF analysis (Figure 3A) demonstrated that treatment with both CoNPs (*p* < 0.001) and SeMet (*p* < 0.01) increased the γH2AX IF intensity. Interestingly, SeMet did not exhibit a synergistic effect with CoNPs with respect to the increase in γH2AX IF intensity. Pretreatment with SeMet attenuated the CoNP-induced increase in γH2AX IF intensity (*p* < 0.05).

The comet assay was performed and we measured 8-OHdG levels in cells to comprehensively evaluate DNA damage. CoNPs notably increased the comet tail length (*p* < 0.001, Figure 3B) and 8-OHdG level (*p* < 0.001, Figure 3C). Although the γH2AX level was increased by SeMet, it did not increase the comet tail length or 8-OHdG level. Moreover, SeMet suppressed the effects of CoNPs on increasing the comet tail length (*p* < 0.01) and 8-OHdG level (*p* < 0.05).

**Figure 4:**
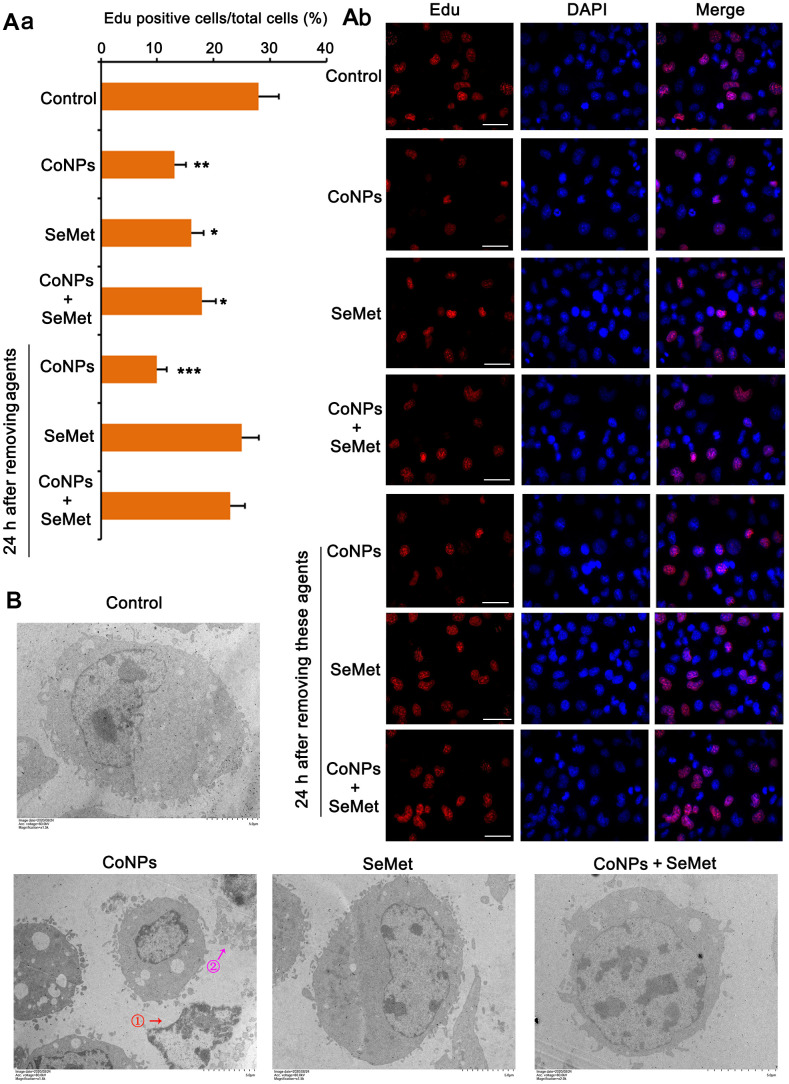
**Cell damage was evaluated by cell proliferation assay and electron microscope observation.** (**A**) CD34^+^ HSC/HPCs were treated with 10 μM SeMet for 15 h or 200 μM CoNPs for 24 h. Alternatively, CD34^+^ HSC/HPCs were treated with 10 μM SeMet for 15 h before additional treatment with 200 μM CoNPs for 24 h. Edu staining was performed immediately after these treatments were finished. In addition, after these treatments were finished, cells were culture in fresh medium for 24 h before Edu staining. (**B**) CD34^+^ HSC/HPCs were treated with 10 μM SeMet and 200 μM CoNPs, alone or in combination, followed by electron microscope observation. *p < 0.05, **p < 0.01, and ***p < 0.001 vs. control cells that did no subjected to any treatments.

Activation of DNA damage response signals induces cell cycle arrest. This can temporarily inhibit cell proliferation so that cells have more time to repair damaged DNA and avoid passing on this damage to their daughter cells. Therefore, we performed the EdU staining assay to examine whether cell proliferation was affected upon the activation of the DNA damage response signal. The results showed that treatment with CoNPs (*p* < 0.01), SeMet (*p* < 0.05), and SeMet + CoNPs (*p* < 0.05) suppressed cell proliferation (Figure 4A). Cell proliferation was still inhibited 24 h after CoNPs were removed from the cells; however, cell proliferation was restored 24 h after SeMet and SeMet + CoNPs were removed.

Using an electron microscope, we observed the changes in the cell ultrastructure (Figure 4B). Treatment with CoNPs caused shrinkage of human CD34^+^ HSCs/HPCs and chromatin (marked by red arrow and the ① symbol in the picture), resulting in cell lysis (marked by a purple arrow and the ② symbol in the picture). SeMet did not cause a notable change in CD34^+^ HSCs/HPCs compared with the control. Furthermore, SeMet hindered CoNP-induced pathological changes in human CD34^+^ HSCs/HPCs.

**Figure 5:**
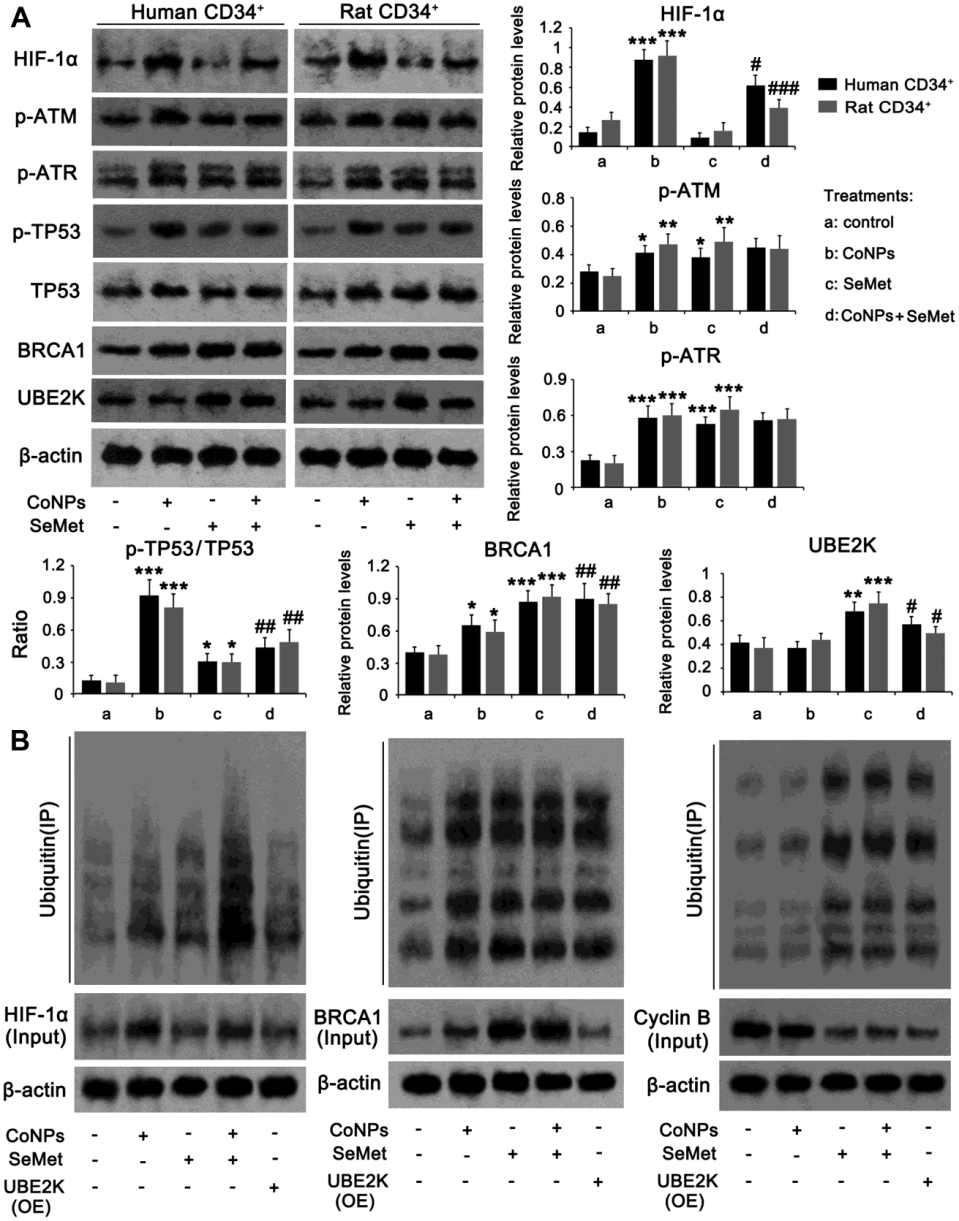
**The regulatory effects of CoNPs and SeMet on DNA damage response signal.** CD34+ HSC/HPCs were treated with 10 μM SeMet and 200 μM CoNPs, alone or in combination, followed by western blot (**A**) and Co-IP assay (**B**). In Co-IP assay, HIF-1α, BRCA1 and cyclin B proteins were immunoprecipitated and the enrichment of ubiquitin in these proteins was further determined by western blot. *p < 0.05, **p < 0.01, and ***p < 0.001 vs. control cells that did no subjected to any treatments. #p < 0.05, ##p < 0.01 and ###p < 0.001 vs. cells treated with CoNPs alone.

Based on the different effects of CoNPs and SeMet on DNA damage and cell proliferation, we assessed any differences in their ability to modulate DNA damage response signals. Western blots showed that treatments with CoNPs and SeMet, increased the levels of p-ATM, p-ATR, p-TP53/TP53 ratio, and BRCA1 (Figure 5A). Except for the increased levels of these factors, there was no difference among these treatments. ATM and ATR protein levels were not changed after treatments with CoNPs and SeMet (Supplementary Figure 1A). SeMet attenuated the increase of p-TP53/TP53 ratio that was induced by CoNPs, but further increased BRCA1 protein level in the presence of CoNPs. CoCl_2_ has been confirmed to strongly stimulate the HIF-α signal, which in turn influences DNA damage response signals. SeMet upregulated the expression of an E2 ubiquitin ligase, UBE2K. Ubiquitin is extensively involved in various processes associated with DNA damage response signals. Therefore, we speculated that the regulatory effects of CoNPs and SeMet on this signal were associated with HIF-1α and UBE2K, respectively. CoNPs, but not SeMet, dramatically increased HIF-1α at the protein level (*p* < 0.001). SeMet significantly inhibited the CoNP-induced increase in HIF-1α levels (*p* < 0.05 or *p* < 0.001). SeMet treatment, but not CoNPs, increased the UBE2K protein level (*p* < 0.001). Treatment with SeMet and CoNPs also increased UBE2K protein levels compared to treatment with CoNPs alone (*p* < 0.05).

The HIF-α protein is susceptible to ubiquitin-mediated degradation. UBE2K and BRCA1 serve as E2 and E3 ubiquitin ligases, respectively. Therefore, we analyzed ubiquitin enrichment in HIF-1α and BRCA1 proteins using a Co-IP assay. Despite increased ubiquitin enrichment in the HIF-1α protein resulting from CoNP treatment, there was a more dramatic increase in HIF-1α protein in the CoNP compared to the control group. Thus, CoNPs actually reduced ubiquitin enrichment in the HIF-1α protein (Figure 5B). This suggested that treatment with SeMet alone increased ubiquitination of the HIF-1α protein, whereas SeMet further significantly increased ubiquitination of HIF-1α after CoNP treatment. Overexpression of UBE2K did not influence the HIF-1α protein level significantly nor induce ubiquitination of HIF-1α. Treatment with CoNPs, SeMet, and SeMet + CoNPs not only increased BRCA1 levels, but also increased the ubiquitination of BRCA1. Overexpression of UBE2K did not affect BRCA1 protein levels, but increased the ubiquitination of BRCA1. Cyclin B protein levels are regulated by UBE2K- and BRCA1-mediated ubiquitination. CoNPs marginally decreased cyclin B protein levels, with a moderate effect on the ubiquitination of the cyclin B protein. Treatment with SeMet and SeMet + CoNPs, as well as overexpression of UBE2K, reduced cyclin B protein levels but increased the ubiquitination of cyclin B.

### Suppression of DNA damage response signals attenuated the protective effect of SeMet against CoNPs

**Figure 6:**
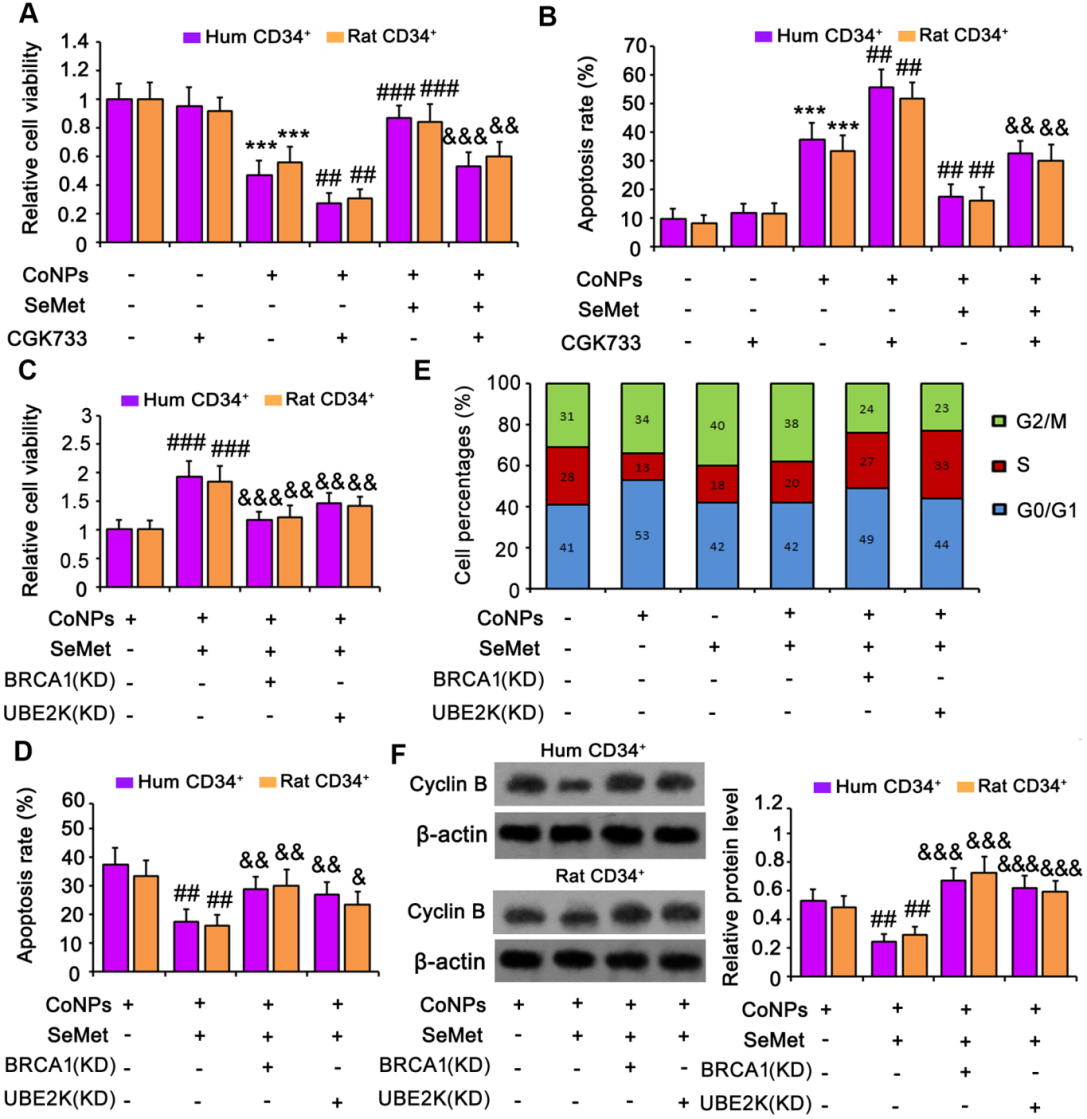
**Suppression of DNA damage response signal attenuated the protection of SeMet against CoNPs.** An inhibitor of ATM/ATR (CGK733) was added to CD34^+^ HSC/HPCs treated with SeMet and CoNPs. Afterwards, cells underwent cell viability (**A**) and apoptosis (**B**) assays. BRCA1 and UBE2K were silenced in CD34^+^ HSC/HPCs, followed by cell viability (**C**), apoptosis (**D**), cell cycle (**E**) and western blot assays (**F**). ***p < 0.001 vs. control cells that did no subjected to any treatments. ^##^*p <* 0.01 and ^###^*p <* 0.001 vs. cells treated with CoNPs alone. ^&^*p <* 0.05, ^&&^*p <* 0.01 and ^&&&^*p <* 0.001 vs. cells treated with SeMet and CoNPs in combination.

An inhibitor of ATM/ATR (CGK733) was added to block activation of DNA damage response signals. Treatment with CGK733 alone, or in combination with SeMet, had no effect on cell viability (Figure 6A) or apoptosis (Figure 6B and Supplementary Figure 1B). However, CGK733 increased the toxicity of CoNPs (*p* < 0.001), and almost abolished the protective effect of SeMet against CoNPs.

To determine whether BRCA1 and UBE2K were involved in the protection against CoNPs, these factors were silenced in CD34^+^ HSCs/HPCs. Knockdown of BRCA1, a key downstream effector of ATM/ATR signaling, abrogated the protection of SeMet against CoNPs, as indicated by cell viability (Figure 6C) and apoptosis (Figure 6D and Supplementary Figure 1C) assays. Although UBE2K is not a downstream target of ATM/ATR signaling, depletion of UBE2K also impaired the protective effect of SeMet. Moreover, silencing either BRCA1 or UBE2K abolished the inhibition of cyclin B by SeMet in CoNP-treated cells (*p* < 0.001, Figure 6F). Treatment with CoNPs increased the percentage of cells in the G0/G1 phase but decreased the percentage in the S phase (Figure 6E and Supplementary Figure 2A). Treatment with SeMet, alone or in combination with CoNPs, increased the percentage of cells in the G2/M phase, whereas it decreased the percentage in the S phase. However, knockdown of BRCA1 or UBE2K reversed the decreased cell percentages in the S phase caused by SeMet and CoNPs.

**Figure 7:**
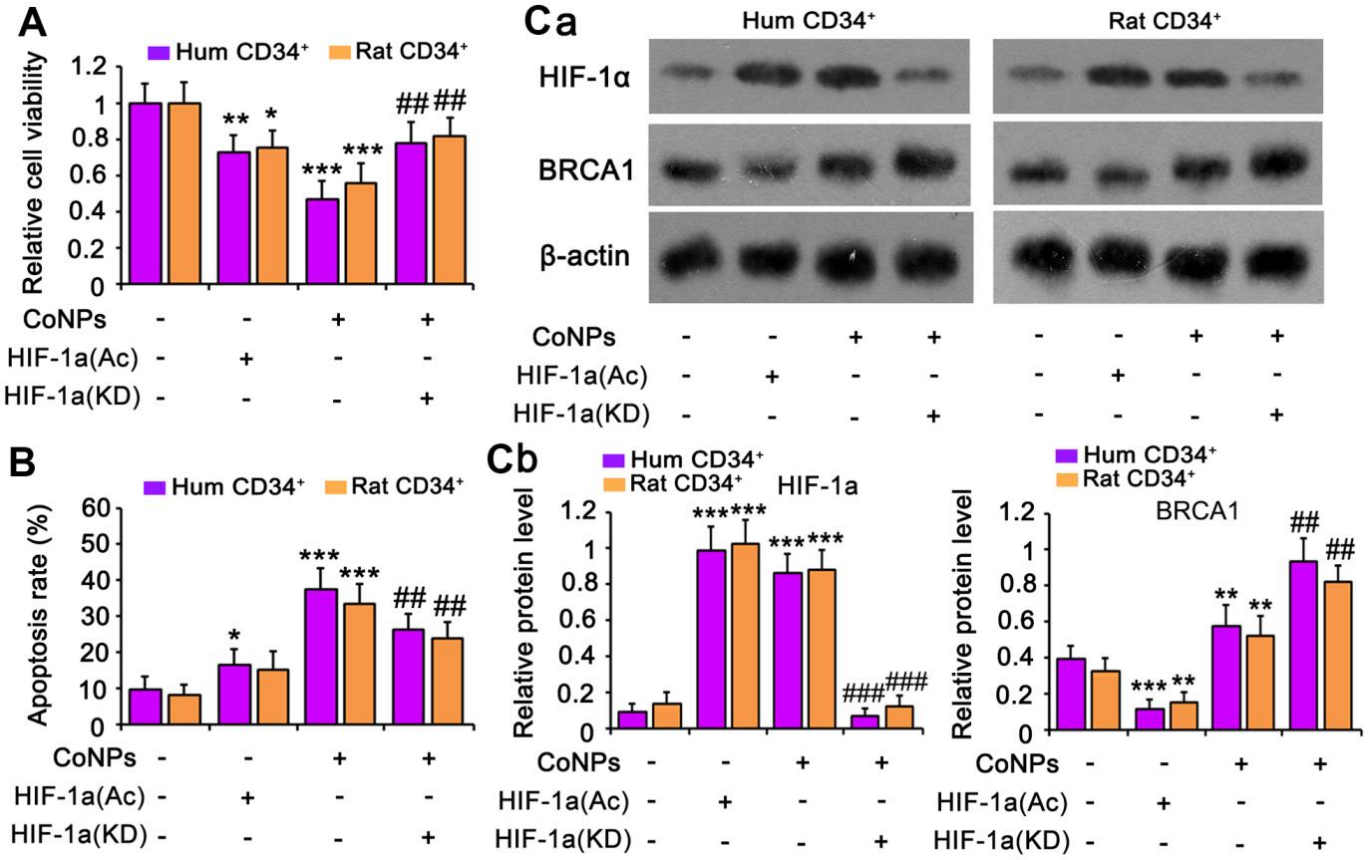
**HIF-1α is implicated in the toxic effect of CoNPs in CD34^+^ HSC/HPCs.** This study used an activator of HIF-1α (DMOG) to imitate the activation of HIF-1α by CoNPs. Furthermore, HIF-1α was knocked down to determine whether HIF-1α partially mediates the toxic effect of CoNPs. After these treatments, cells underwent cell viability (**A**) and apoptosis (**B**) and western blot assays (**C**). *p < 0.05, **p < 0.01 and ***p < 0.001 vs. control cells that did no subjected to any treatments. ^##^*p <* 0.01 and ^###^*p <* 0.001 vs. cells treated with CoNPs alone.

HIF-1α inhibits BRCA1 expression [38, 39]. Although CoNPs promoted BRCA1 expression, we noticed that the promoting effect of CoNPs was weaker than that of SeMet. We used an activator of HIF-1α (dimethyloxalyglycine) to mimic the activation of HIF-1α by CoNPs. The activator increased the expression of HIF-1α but decreased that of BRCA1 (Figure 7C). This resulted in cell damage as indicated by the cell viability (Figure 7A) and apoptosis (Figure 7B and Supplementary Figure 2B) assays. However, the silencing of HIF-1α resulted in attenuation of the toxic effect of CoNPs on CD34^+^ HSCs/HPCs.

### SeMet attenuated the toxic effect of CoNPs on rat CD34^+^ HSCs/HPCs

**Figure 8:**
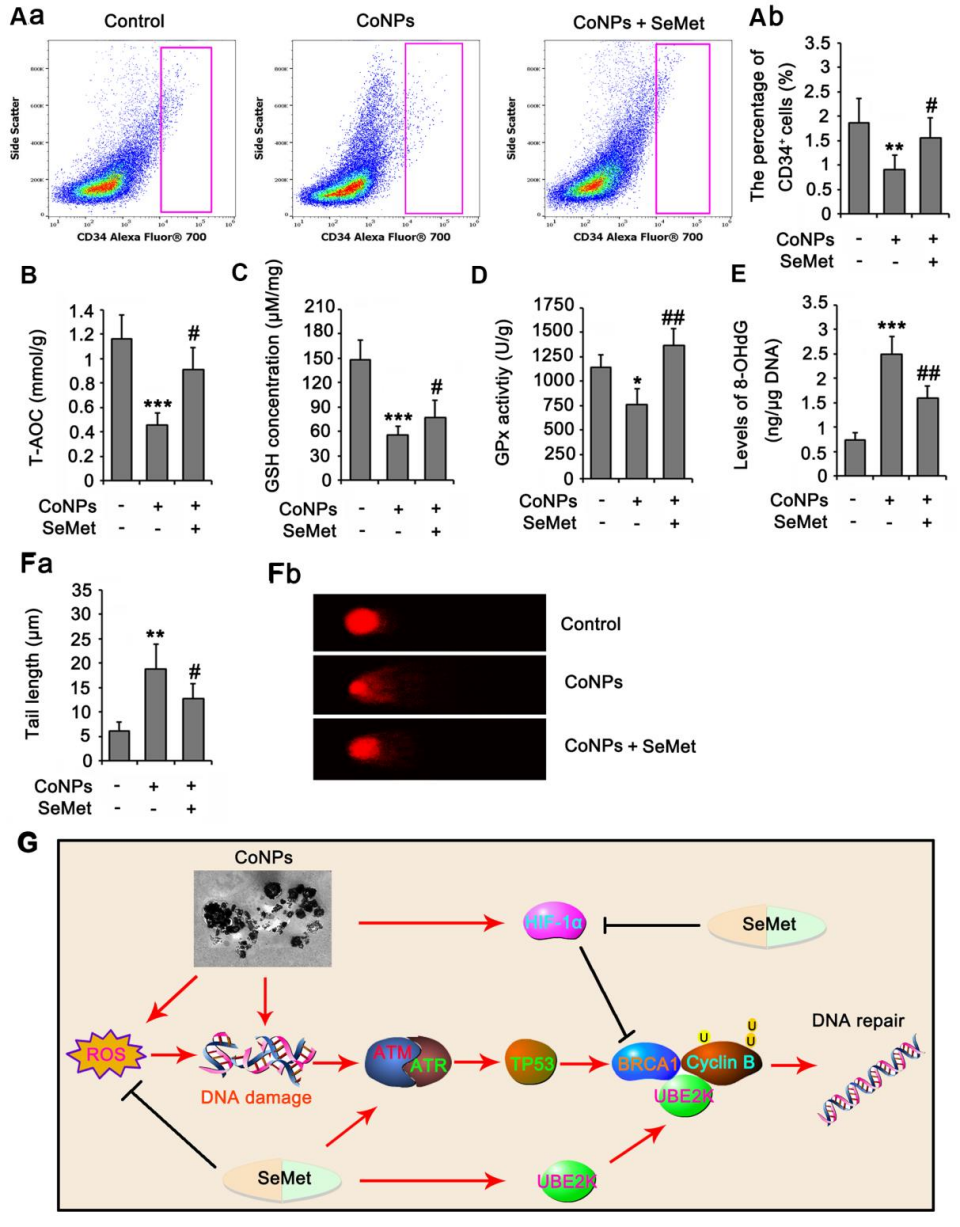
**SeMet attenuated toxic effect of CoNPs on CD34^+^ HSC/HPCs in rat.** Male SD rats were 6-8 weeks old and weighed approximate 800 g at the start of the experiments. CoNPs particles were suspended in a vehicle of 1:1 rat serum: phosphate buffered saline (PBS; Oxoid) by sonication and administered to rats. 50 μL CoNPs (1000 μg/kg BW)-containing vehicle was injected in the right hip joint. The rats were exposed to three injections of the particles at three week intervals. Sham treated rat received 15 ml of vehicle alone. In addition, rats received an oral dose of SeMet (2 mg SeMet/kg BW/day). All rats were sacrificed three weeks after final injection of CoNPs. Bone marrow was collected for analysis of CD34^+^ HSC/HPCs number by flow cytometry (**A**), for the measurements of biochemical parameters, including, T-AOC (**B**), GSH level (**C**), GPx activity (**D**) and 8-OHdG level (**E**), and for comet assay (**F**). (**G**) The mechanism diagram shows the mechanism by which SeMet attenuates toxic effect of CoNPs on CD34^+^ HSC/HPCs. *p < 0.05, **p < 0.01 and ***p < 0.001 vs. control cells that did no subjected to any treatments. ^##^*p <* 0.01 and ^###^*p <* 0.001 vs. cells treated with CoNPs alone.

In animal study, as indicated by flow cytometry assay, the number of rat CD34^+^ HSC/HPCs in bone marrow was reduced by CoNPs (*p*<0.01, Figure 8A), but the reduction was partially reversed by SeMet (*p*<0.05 vs. CoNPs group). The number of red blood cells in peripheral blood was not changed, but the MCV and HCT% were increased after the injection of CoNPs. The total WBC and some types of WBC, such as LYMN, OTHR, and EO, in peripheral blood were also increased after the injection of CoNPs. SeMet partially suppressed the increase of these WBC (Table 1). SeMet attenuated the reduction of T-AOC (*p* < 0.05 vs. CoNPs group, Figure 8B), GSH (*p* < 0.05 vs. CoNPs group, Figure 8C) and GPx activity (*p* < 0.01 vs. CoNPs group, Figure 8D) caused by CoNPs. Long term exposure to CoNPs also caused the DNA damage in rat CD34^+^ HSC/HPCs, based on the increase of 8-OHdG level (*p* < 0.001, Figure 8E) and the tail length (*p* < 0.01, Figure 8F) in comet assay after CoNPs treatment. The CoNPs-induced DNA damage was relieved by SeMet.

**Table 1:** The results from blood routine examination after treatments with SeMet and CoNPs (n=6).

**Items**	**Treatment groups**			**Normal range**
	**Control**	**CoNPs**	**SeMet+CoNPs**	
WBC(10^9^/L)	12	28.8***	**18.5^###^**	3.0-15.0
LYMN(10^9^/L)	7.8	12**	8.4^##^	4.0-10.0
OTHR(10^9^/L)	0.2	10.2***	7^#^	0.0-0.2
EO(10^9^/L)	4	**5.7**	3.1	1.0-4.0
LYMN%	65	41.6**	45.4	40.0-95.0
OTHR%	1.6	35.4***	**37.3**	0.0-14.0
EO%	33.3	19.8**	16.8	4.0-50.0
RBC(10^12^/L)	7.8	10.6	8.31	5.0-12.0
HGB(g/L)	170	163	152	111-180
MCV(fL)	62	88.4*	**86.4**	44.5-69.0
MCH(pg)	21.8	23.2	22.6	12.0-24.5
MCHC(g/L)	255	295	273	216-420
RDW-CV	14.9	15.3	13.3	12.0-27.0
RDW-SD	49	54	45	25.0-70.0
HCT%	51.9	93.7***	**71.8^#^**	36.0-52.0
PLT(10^9^/L)	236	202*	245^#^	140-600
MPV(fL)	9.7	10.2	9.8	5.0-20.0
PDW	14.1	14.8	14.1	8.0-18.0
PCT%	0.25	0.21	0.26	0.1-0.35

WBC: white blood cells; LYMN: lymphocytes; OTHR: neutrophils; EO: eosinophils; LYMN%= LYMN/WBC×100%; OTHR%= OTHR/WBC×100%; EO%= EO/WBC×100%; RBC: red blood cells; HGB: hemoglobin; MCV: mean corpuscular volume; MCH: mean corpuscular hemoglobin; MCHC: mean corpuscular hemoglobin concentration; RDW-CV: mean red blood cell distribution width-coefficient of variation; RDW-SD: red blood cell distribution width; HCT: hematocrit; PLT: platelet counts, MPV: mean platelet volume; PDW: platelet distribution width. *p < 0.05, **p < 0.01, and ***p < 0.001 vs. control cells that did no subjected to any treatments; ^#^*p* < 0.05, ^##^*p* < 0.01 and ^###^*p* < 0.001 vs. cells treated with CoNPs alone. Bold font indicates that the data are out of the normal ranges.

## DISCUSSION

CoNPs cause overproduction of ROS, resulting in oxidative damage to many cellular components [6]. The current study also found an increase in ROS production as well as DNA damage in CoNP-treated CD34^+^ HSCs/HPCs. This DNA damage was probably associated with the increase in ROS because 8-OHdG, the major oxidative product of DNA, was increased after CoNP treatment. However, some studies reported that supplementation with antioxidants cannot attenuate the toxic effect of CoNPs completely, and heavy metals themselves cause DNA damage by breaking the intermolecular bonds [7, 28, 29].

Normal hematopoiesis is a strictly regulated process that is achieved through the self-renewal of HSCs, proliferation of lineage-committed HPCs, and maturation of differentiated cells. Previous studies reported the toxic effect of CoNPs on HSCs and HPCs *in vitro* [12], but the potential consequences of exposure to CoNPs generated by THR have not been adequately investigated *in vivo.* This study showed that CoNPs have a detrimental effect on CD34^+^ HSCs/HPCs, not only *in vitro* but also in a rat model. Long-term exposure to CoNPs increased ROS levels and caused DNA damage due to the reduction of CD34^+^ HSCs/HPCs in bone marrow. An interesting phenomenon observed in this study was that CoNPs did not decrease the number of erythrocytes in peripheral blood. This result is probably due to the strong proliferative capacity of HPCs, which can compensate for their loss caused by CoNPs. Surprisingly, CoNPs increased the number of lymphocytes, neutrophils, and eosinophils in peripheral blood. A previous study reported that CoNPs triggered an inflammatory response [7], which may stimulate the differentiation of CD34^+^ HSC/HPCs to these inflammatory cells or their aberrant proliferation.

Although Se has been confirmed to have multiple beneficial functions, there are reports of Se toxicity at high doses [17]. In this study, three types of Se-containing substances (Na_2_SeO_3_, SeMet, and SeCys) were used to determine their effects on CoNP-induced toxicity. All three Se-containing substances showed similar protection against CoNPs, suggesting that the protective effect is primarily dependent on Se, rather than whether the Se is organic or inorganic. The concentrations of Se that showed the optimal protective effect differed between organic and inorganic Se. The optimal concentration of SeMet was much higher than that of Na_2_SeO_3_, and similar to the minimum toxic dose. Therefore, SeMet might be safer for practical use in attenuating the toxicity of CoNPs. Se is known for its antioxidant capacity. Gunes found that supplementation with 1 mg/kg Se was more effective at increasing the GSH level than 0.5 mg/kg Se during cyclophosphamide-induced cardiotoxicity in rats [30]. Se increases the production of selenoproteins, most of which play important roles in antioxidative functions. The present study showed that SeMet improved the antioxidant/oxidant balance that was impaired by CoNPs. However, after abolishing the improvement of antioxidant function by H_2_O_2_, SeMet still displayed a protective effect against CoNPs, which implied another mechanism underlying its protection.

This study found that SeMet stimulated DNA damage response signals because signaling molecules, such as p-ATM, p-ATR, γH2AX, p-p53, p53 and BRCA1, were increased by SeMet in the presence or absence of CoNPs. However, SeMet probably did not cause DNA damage according to the results of the comet assay, 8-OHdG measurement, and electron microscopy. Moreover, when SeMet was removed from the culture medium, the proliferation of CD34^+^ HSCs/HPCs was restored. This also suggested that CD34^+^ HSCs/HPCs did not suffer from DNA damage.

Se can protect DNA from damage by activating redox factor-1 (Ref1)/p53. Ref1 binds to p53, promotes p53 tetramerization, and enhances p53 sequence-specific DNA binding in its reduced state [31–33]. p53 is well-known for its pro-apoptotic role in cancer prevention, but p53 also plays an essential role in DNA repair pathways because of its association with BRCA1, APE1, and Gadd45a proteins [30]. Se has also been reported to activate ATM/ATR, but this activation is associated with Se-induced DNA damage in cancer cells [34]. However, Seo et al. found that SeMet, even at a high concentration (100 μM), did not induce DNA damage, but protected cells from DNA damage caused by ultraviolet rays by activating DNA damage response signals [35]. Our results, in line with this finding, suggest that Se can activate DNA damage response signals without inducing DNA damage.

CoNPs also activated DNA damage response signals by increasing the levels of signaling molecules with SeMet. In contrast, CoNPs led to severe DNA damage, and the suppressed proliferation was not recovered even after removing CoNPs. We speculate that activation of the DNA damage response signals is the result of DNA damage caused by CoNPs. DNA damage is a detrimental lesion in cells. If damaged DNA is unrepaired, DNA damage response signals can finally switch from survival to apoptosis signals to induce cell death. In this process, p53 also plays an important role [36]. Therefore, the role of DNA damage response signals induced by CoNPs in cell survival or apoptosis needs further elucidation.

An interesting phenomenon is that the activation of DNA damage response signals by SeMet can effectively prevent DNA damage caused by CoNPs. One possible reason is that activation of these signals in advance is more effective than activation after DNA damage has occurred. A previous study suggested that the induction of DNA repair and DNA damage protection required a 15-h pretreatment with SeMet [35]. Neither DNA repair nor protection was observed when SeMet was administered concurrently with DNA damage [32, 35]. In addition, SeMet induced a more dramatic increase in BRCA1 than CoNPs. BRCA1 participates in numerous cellular processes essential for maintaining genomic integrity, such as regulation of cell cycle checkpoint control, homologous recombination and DNA repair, centrosome amplification, transcription, and chromatin dynamics [37]. CoNPs are a strong activator of HIF-1α that has an inhibitory effect on BRCA1 [38, 39]. Thus, CoNP-induced HIF-1α likely attenuated the increase in BRCA1 due to activation of DNA damage response signals. Indeed, silencing HIF-1α promoted the increase in BRCA1 after CoNP treatment, and attenuated the toxicity of CoNPs. Conversely, SeMet inhibited HIF-1α probably via the following two mechanisms: 1) Se binding protein-1 (hSP56) is a negative regulator of HIF-1α [40], and 2) SeMet lowered intracellular ROS, which disrupts the ubiquitin-mediated degradation of HIF-1α [41].

This study showed that SeMet not only upregulated BRCA1, but also enhanced its function by elevating UBE2K. BRCA1 is an ubiquitin E3 ligase enzyme and the BRCA1-mediated ubiquitination modification, especially at the K63 site in proteins, is critical for the activation of DNA damage response signals [42, 43]. This ubiquitination modification at K63 does not induce protein degradation, but promotes signal activation similar to phosphorylation modification. For example, the BRCA1–BARD1 heterodimer constitutes an additional source of H2A ubiquitination and activation in the DNA repair response [37]. However, UBE2K, when linked to BRCA1, only promotes its role in ubiquitination modification at the K48 site in proteins [21]. Ubiquitination at the K48 site is likely to induce protein degradation. UBE2K, together with BRCA1, induces ubiquitination of cyclin B at the K48 site and consequent protein degradation. The current study observed that silencing either BRCA1 or UBE2K disrupted the cell cycle arrest induced by SeMet, with loss of the protective effect of SeMet against CoNPs. This result suggested that UBE2K/BRCA1-mediated ubiquitination at K48 is also critical for the DNA damage response signal and SeMet protection. A previous study found that pre-activation of the genome integrity checkpoint increases cell tolerance to DNA damage [44].

It should be noted that both CoNPs and SeMet increased p-ATM and p-ATR protein levels, but the underlying mechanisms were different. CoNPs induced DNA damage, which activated ATM/ATR. SeMet did not cause DNA damage, thus SeMet activated ATM/ATR probably through other mechanism. Although CoNPs-induced DNA damage activated ATM/ATR pathway. Activation of the pathway, especially the downstream effecter BCRA1, was attenuated by HIF-1α that was upregulated by CoNPs as well. Differently, SeMet not only activated ATM/ATR pathway, but also increased BCRA1 function by upregulating UBE2K. Therefore, SeMet promoted DNA repair by activating ATM/ATR pathway, blockage the pathway by CGK733 attenuated the protective effect of SeMet against CoNPs.

In summary, SeMet protected CD34^+^ HSCs/HPCs from CoNPs by elevating their antioxidative capacity and activating DNA damage response signals. As shown in Figure 8G, both SeMet and CoNPs stimulated the DNA damage response signals, but the former did not cause DNA damage. SeMet inhibited the CoNP-induced increase in HIF-1α, thereby disrupting the inhibitory effect of HIF-1α on BRCA1. Moreover, SeMet promoted BRCA1-mediated ubiquitination of cyclin B at K48 by upregulating UBE2K. Therefore, SeMet enhanced cell cycle arrest and DNA repair after exposure to CoNPs. This study reveals a novel protective mechanism underlying the protective effect of SeMet against CoNPs.

## Supplementary Material

Supplementary Figures

## References

[1] Sabbioni E, Fortaner S, Farina M, Del Torchio R, Olivato I, Petrarca C, Bernardini G, Mariani-Costantini R, Perconti S, Di Giampaolo L, Gornati R, Di Gioacchino M. Cytotoxicity and morphological transforming potential of cobalt nanoparticles, microparticles and ions in Balb/3T3 mouse fibroblasts: an *in vitro* model. Nanotoxicology. 2014; 8:455–64. 10.3109/17435390.2013.796538 23586465

[2] Colognato R, Bonelli A, Ponti J, Farina M, Bergamaschi E, Sabbioni E, Migliore L. Comparative genotoxicity of cobalt nanoparticles and ions on human peripheral leukocytes *in vitro*. Mutagenesis. 2008; 23:377–82. 10.1093/mutage/gen024 18504271

[3] Laovitthayanggoon S, Henderson CJ, McCluskey C, MacDonald M, Tate RJ, Grant MH, Currie S. Cobalt administration causes reduced contractility with parallel increases in TRPC6 and TRPM7 transporter protein expression in adult rat hearts. Cardiovasc Toxicol. 2019; 19:276–86. 10.1007/s12012-018-9498-3 30523498 PMC6505488

[4] Liu YK, Deng XX, Yang HL. Cytotoxicity and genotoxicity in liver cells induced by cobalt nanoparticles and ions. Bone Joint Res. 2016; 5:461–69. 10.1302/2046-3758.510.BJR-2016-0016.R1 27754833 PMC5075796

[5] Zheng F, Luo Z, Zheng C, Li J, Zeng J, Yang H, Chen J, Jin Y, Aschner M, Wu S, Zhang Q, Li H. Comparison of the neurotoxicity associated with cobalt nanoparticles and cobalt chloride in Wistar rats. Toxicol Appl Pharmacol. 2019; 369:90–99. 10.1016/j.taap.2019.03.003 30849457

[6] Liu Y, Yang X, Wang W, Wu X, Zhu H, Liu F. Melatonin counteracts cobalt nanoparticle-induced cytotoxicity and genotoxicity by deactivating reactive oxygen species-dependent mechanisms in the NRK cell line. Mol Med Rep. 2017; 16:4413–20. 10.3892/mmr.2017.7309 28849220 PMC5647000

[7] Nyga A, Hart A, Tetley TD. Importance of the HIF pathway in cobalt nanoparticle-induced cytotoxicity and inflammation in human macrophages. Nanotoxicology. 2015; 9:905–17. 10.3109/17435390.2014.991430 25676618

[8] Yan J, Tie G, Wang S, Tutto A, DeMarco N, Khair L, Fazzio TG, Messina LM. Diabetes impairs wound healing by Dnmt1-dependent dysregulation of hematopoietic stem cells differentiation towards macrophages. Nat Commun. 2018; 9:33. 10.1038/s41467-017-02425-z 29295997 PMC5750226

[9] Choi J, Baldwin TM, Wong M, Bolden JE, Fairfax KA, Lucas EC, Cole R, Biben C, Morgan C, Ramsay KA, Ng AP, Kauppi M, Corcoran LM, et al. Haemopedia RNA-seq: a database of gene expression during haematopoiesis in mice and humans. Nucleic Acids Res. 2019; 47:D780–85. 10.1093/nar/gky1020 30395284 PMC6324085

[10] Leins H, Mulaw M, Eiwen K, Sakk V, Liang Y, Denkinger M, Geiger H, Schirmbeck R. Aged murine hematopoietic stem cells drive aging-associated immune remodeling. Blood. 2018; 132:565–76. 10.1182/blood-2018-02-831065 29891535 PMC6137572

[11] Li XL, Xue Y, Yang YJ, Zhang CX, Wang Y, Duan YY, Meng YN, Fu J. Hematopoietic stem cells: cancer involvement and myeloid leukemia. Eur Rev Med Pharmacol Sci. 2015; 19:1829–36. 26044227

[12] Bregoli L, Chiarini F, Gambarelli A, Sighinolfi G, Gatti AM, Santi P, Martelli AM, Cocco L. Toxicity of antimony trioxide nanoparticles on human hematopoietic progenitor cells and comparison to cell lines. Toxicology. 2009; 262:121–29. 10.1016/j.tox.2009.05.017 19482055

[13] Rona G, Pagano M. Mixed ubiquitin chains regulate DNA repair. Genes Dev. 2019; 33:1615–16. 10.1101/gad.334383.119 31792015 PMC6942041

[14] Schwertman P, Bekker-Jensen S, Mailand N. Regulation of DNA double-strand break repair by ubiquitin and ubiquitin-like modifiers. Nat Rev Mol Cell Biol. 2016; 17:379–94. 10.1038/nrm.2016.58 27211488

[15] Huen MS, Grant R, Manke I, Minn K, Yu X, Yaffe MB, Chen J. RNF8 transduces the DNA-damage signal via histone ubiquitylation and checkpoint protein assembly. Cell. 2007; 131:901–14. 10.1016/j.cell.2007.09.041 18001825 PMC2149842

[16] Brozmanová J, Mániková D, Vlčková V, Chovanec M. Selenium: a double-edged sword for defense and offence in cancer. Arch Toxicol. 2010; 84:919–38. 10.1007/s00204-010-0595-8 20871980

[17] Valdiglesias V, Pásaro E, Méndez J, Laffon B. *In vitro* evaluation of selenium genotoxic, cytotoxic, and protective effects: a review. Arch Toxicol. 2010; 84:337–51. 10.1007/s00204-009-0505-0 20033805

[18] de Rosa V, Erkekoğlu P, Forestier A, Favier A, Hincal F, Diamond AM, Douki T, Rachidi W. Low doses of selenium specifically stimulate the repair of oxidative DNA damage in LNCaP prostate cancer cells. Free Radic Res. 2012; 46:105–16. 10.3109/10715762.2011.647009 22145923 PMC3332102

[19] Favrot C, Beal D, Blouin E, Leccia MT, Roussel AM, Rachidi W. Age-dependent protective effect of selenium against UVA irradiation in primary human keratinocytes and the associated DNA repair signature. Oxid Med Cell Longev. 2018; 2018:5895439. 10.1155/2018/5895439 29682159 PMC5842700

[20] Fu L, Yan X, Ruan X, Lin J, Wang Y. Differential protein expression of Caco-2 cells treated with selenium nanoparticles compared with sodium selenite and selenomethionine. Nanoscale Res Lett. 2014; 9:589. 10.1186/1556-276X-9-589 25426004 PMC4241056

[21] Christensen DE, Brzovic PS, Klevit RE. E2-BRCA1 RING interactions dictate synthesis of mono- or specific polyubiquitin chain linkages. Nat Struct Mol Biol. 2007; 14:941–48. 10.1038/nsmb1295 17873885

[22] Shabbeer S, Omer D, Berneman D, Weitzman O, Alpaugh A, Pietraszkiewicz A, Metsuyanim S, Shainskaya A, Papa MZ, Yarden RI. BRCA1 targets G2/M cell cycle proteins for ubiquitination and proteasomal degradation. Oncogene. 2013; 32:5005–16. 10.1038/onc.2012.522 23246971 PMC3796024

[23] Kleiman FE, Wu-Baer F, Fonseca D, Kaneko S, Baer R, Manley JL. BRCA1/BARD1 inhibition of mRNA 3’ processing involves targeted degradation of RNA polymerase II. Genes Dev. 2005; 19:1227–37. 10.1101/gad.1309505 15905410 PMC1132008

[24] Brown C, Lacharme-Lora L, Mukonoweshuro B, Sood A, Newson RB, Fisher J, Case CP, Ingham E. Consequences of exposure to peri-articular injections of micro- and nano-particulate cobalt-chromium alloy. Biomaterials. 2013; 34:8564–80. 10.1016/j.biomaterials.2013.07.073 23932295

[25] Chattopadhyay S, Dash SK, Tripathy S, Das B, Mandal D, Pramanik P, Roy S. Toxicity of cobalt oxide nanoparticles to normal cells; an *in vitro* and *in vivo* study. Chem Biol Interact. 2015; 226:58–71. 10.1016/j.cbi.2014.11.016 25437709

[26] Sakamoto M, Yasutake A, Kakita A, Ryufuku M, Chan HM, Yamamoto M, Oumi S, Kobayashi S, Watanabe C. Selenomethionine protects against neuronal degeneration by methylmercury in the developing rat cerebrum. Environ Sci Technol. 2013; 47:2862–68. 10.1021/es304226h 23398308

[27] Krittaphol W, Wescombe PA, Thomson CD, McDowell A, Tagg JR, Fawcett JP. Metabolism of L-selenomethionine and selenite by probiotic bacteria: *in vitro* and *in vivo* studies. Biol Trace Elem Res. 2011; 144:1358–69. 10.1007/s12011-011-9057-2 21494803

[28] Hartwig A, Asmuss M, Blessing H, Hoffmann S, Jahnke G, Khandelwal S, Pelzer A, Bürkle A. Interference by toxic metal ions with zinc-dependent proteins involved in maintaining genomic stability. Food Chem Toxicol. 2002; 40:1179–84. 10.1016/s0278-6915(02)00043-1 12067581

[29] Porchetta A, Vallée-Bélisle A, Plaxco KW, Ricci F. Allosterically tunable, DNA-based switches triggered by heavy metals. J Am Chem Soc. 2013; 135:13238–41. 10.1021/ja404653q 23971651 PMC3831027

[30] Gunes S, Sahinturk V, Karasati P, Sahin IK, Ayhanci A. Cardioprotective effect of selenium against cyclophosphamide-induced cardiotoxicity in rats. Biol Trace Elem Res. 2017; 177:107–14. 10.1007/s12011-016-0858-1 27709497

[31] Fischer JL, Lancia JK, Mathur A, Smith ML. Selenium protection from DNA damage involves a Ref1/p53/Brca1 protein complex. Anticancer Res. 2006; 26:899–904. 16619485

[32] Seo YR, Kelley MR, Smith ML. Selenomethionine regulation of p53 by a ref1-dependent redox mechanism. Proc Natl Acad Sci USA. 2002; 99:14548–53. 10.1073/pnas.212319799 12357032 PMC137920

[33] Seemann S, Hainaut P. Roles of thioredoxin reductase 1 and APE/Ref-1 in the control of basal p53 stability and activity. Oncogene. 2005; 24:3853–63. 10.1038/sj.onc.1208549 15824742

[34] Qi Y, Schoene NW, Lartey FM, Cheng WH. Selenium compounds activate ATM-dependent DNA damage response via the mismatch repair protein hMLH1 in colorectal cancer cells. J Biol Chem. 2010; 285:33010–17. 10.1074/jbc.M110.137406 20709753 PMC2963351

[35] Seo YR, Sweeney C, Smith ML. Selenomethionine induction of DNA repair response in human fibroblasts. Oncogene. 2002; 21:3663–69. 10.1038/sj.onc.1205468 12032834

[36] Williams AB, Schumacher B. P53 in the DNA-damage-repair process. Cold Spring Harb Perspect Med. 2016; 6:a026070. 10.1101/cshperspect.a026070 27048304 PMC4852800

[37] Kalb R, Mallery DL, Larkin C, Huang JT, Hiom K. BRCA1 is a histone-H2A-specific ubiquitin ligase. Cell Rep. 2014; 8:999–1005. 10.1016/j.celrep.2014.07.025 25131202 PMC4382519

[38] Koshiji M, Kageyama Y, Pete EA, Horikawa I, Barrett JC, Huang LE. HIF-1alpha induces cell cycle arrest by functionally counteracting Myc. EMBO J. 2004; 23:1949–56. 10.1038/sj.emboj.7600196 15071503 PMC404317

[39] Manalo DJ, Rowan A, Lavoie T, Natarajan L, Kelly BD, Ye SQ, Garcia JG, Semenza GL. Transcriptional regulation of vascular endothelial cell responses to hypoxia by HIF-1. Blood. 2005; 105:659–69. 10.1182/blood-2004-07-2958 15374877

[40] Jeong JY, Zhou JR, Gao C, Feldman L, Sytkowski AJ. Human selenium binding protein-1 (hSP56) is a negative regulator of HIF-1α and suppresses the malignant characteristics of prostate cancer cells. BMB Rep. 2014; 47:411–16. 10.5483/bmbrep.2014.47.7.104 24874852 PMC4163856

[41] Jung SN, Yang WK, Kim J, Kim HS, Kim EJ, Yun H, Park H, Kim SS, Choe W, Kang I, Ha J. Reactive oxygen species stabilize hypoxia-inducible factor-1 alpha protein and stimulate transcriptional activity via AMP-activated protein kinase in DU145 human prostate cancer cells. Carcinogenesis. 2008; 29:713–21. 10.1093/carcin/bgn032 18258605

[42] Zhu B, Yan K, Li L, Lin M, Zhang S, He Q, Zheng D, Yang H, Shao G. K63-linked ubiquitination of FANCG is required for its association with the Rap80-BRCA1 complex to modulate homologous recombination repair of DNA interstand crosslinks. Oncogene. 2015; 34:2867–78. 10.1038/onc.2014.229 25132264

[43] Liu P, Gan W, Su S, Hauenstein AV, Fu TM, Brasher B, Schwerdtfeger C, Liang AC, Xu M, Wei W. K63-linked polyubiquitin chains bind to DNA to facilitate DNA damage repair. Sci Signal. 2018; 11:eaar8133. 10.1126/scisignal.aar8133 29871913 PMC6434707

[44] Tsaponina O, Chabes A. Pre-activation of the genome integrity checkpoint increases DNA damage tolerance. Nucleic Acids Res. 2013; 41:10371–78. 10.1093/nar/gkt820 24049076 PMC3905891

