# Supplementary material for: Selenomethionine protects hematopoietic stem/progenitor cells against cobalt nanoparticles by stimulating antioxidant actions and DNA repair functions
Source: Aging (Albany NY). 2021 Apr 19;13(8):11705–26. doi: 10.18632/aging.202865 (PMC8109066; doi:10.18632/aging.202865)
Supplement: Supplementary Figures [file aging-13-202865-s001.pdf]

SUPPLEMENTARY FIGURES

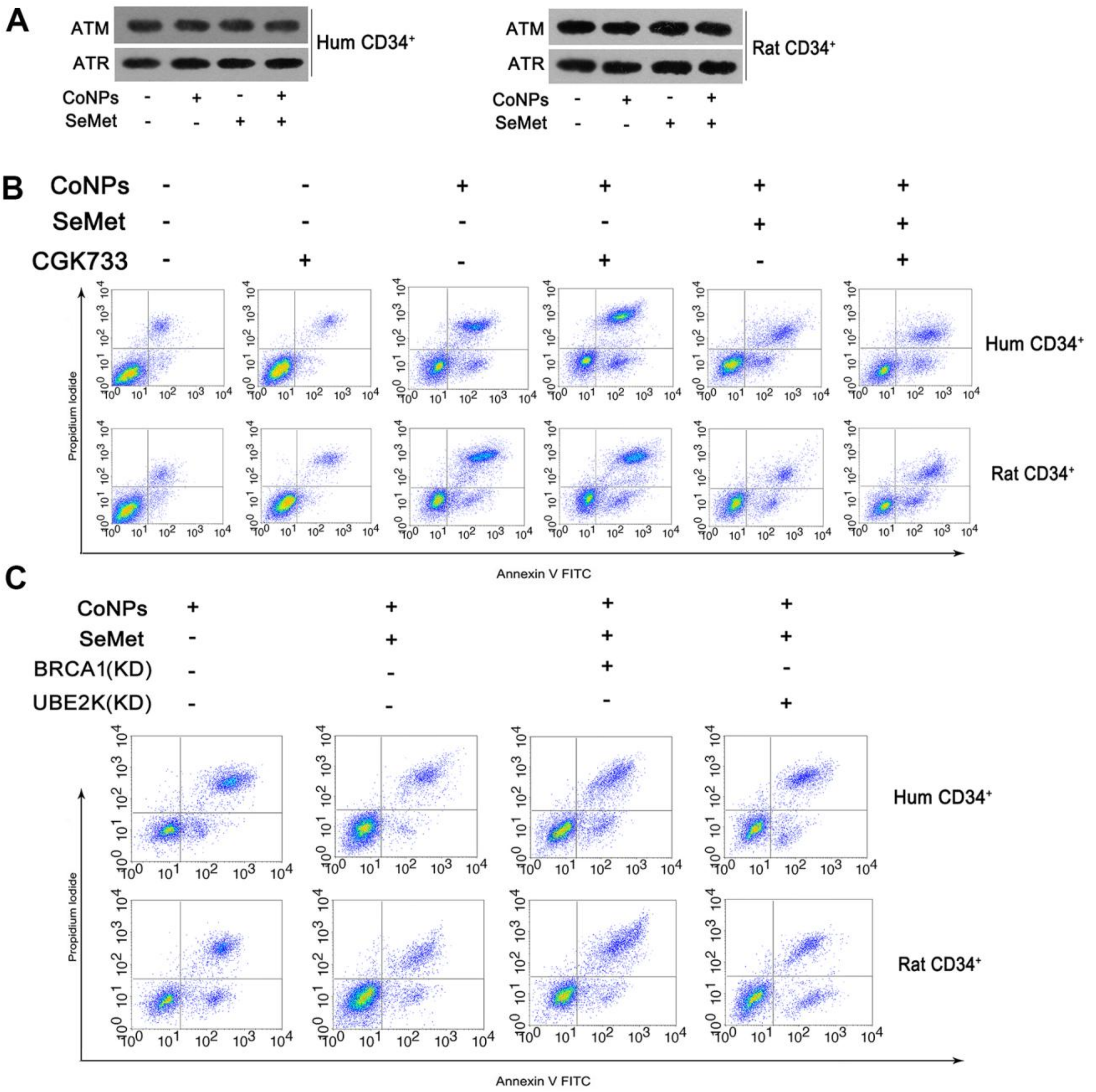

Supplementary Figure 1. The protective effects of SeMet are through modulating ATM/ATR and HIF-1α. (**A**) Western blot was conducted to detect ATM and ATR protein levels after the treatments with CoNPs and SeMet. (**B**) The apoptosis rate after treatments with CoNPs, SeMet and CGK733. (**C**) The apoptosis rate after treatments with CoNPs and SeMet, or knockdown of BRCA1 and UBE2K.

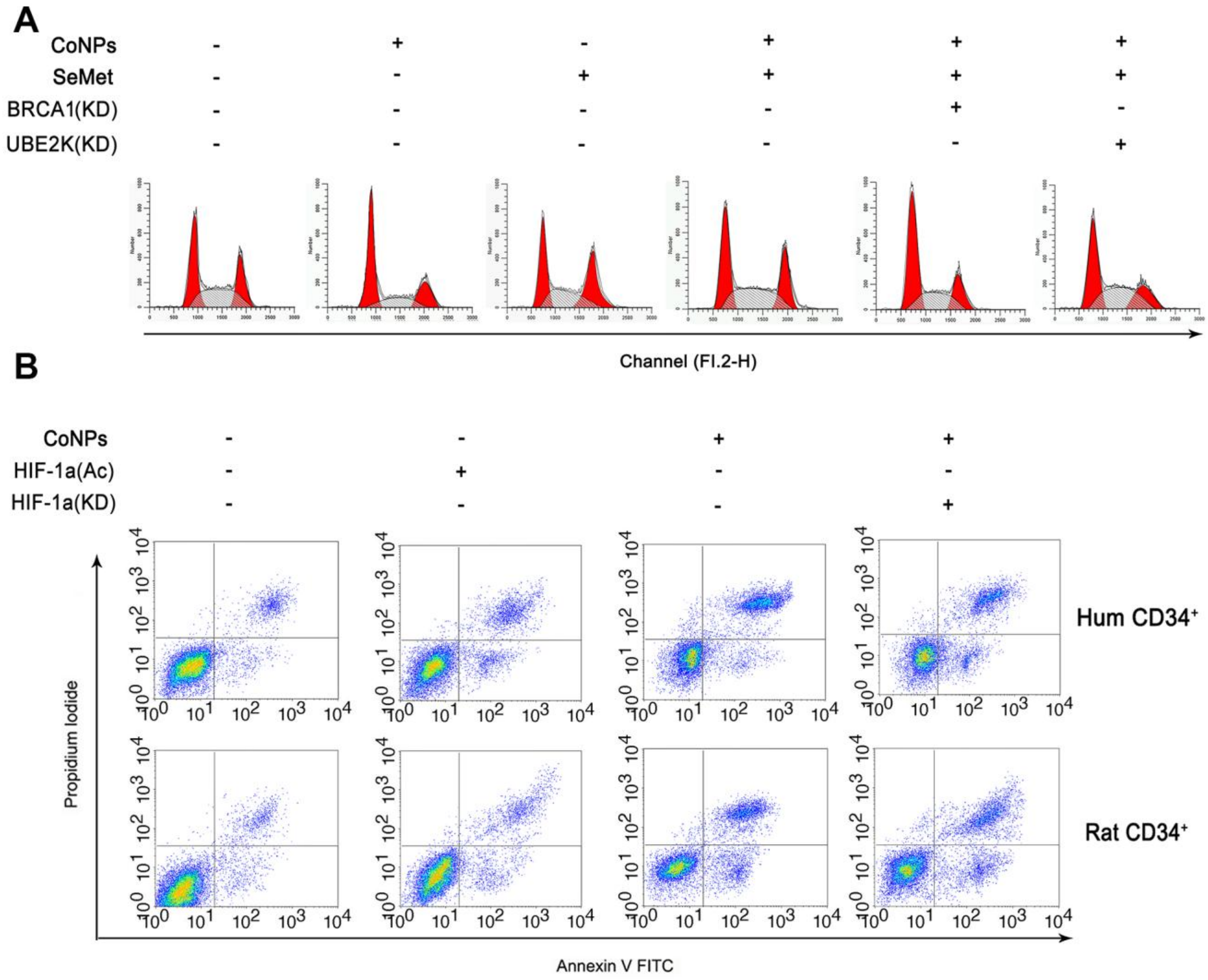

Supplementary Figure 2. BRCA1 and HIF-1α mediated the toxicity of CoNPs and the protection of SeMet. (**A**) the alteration of cell cycle after treatments with CoNPs and SeMet, or knockdown of BRCA1 and UBE2K. (**B**) The apoptosis rate after treatments with CoNPs and HIF-1α activator, or knockdown of HIF-1α.
